# Prediction of Effect of Pegylated Interferon Alpha-2b plus Ribavirin Combination Therapy in Patients with Chronic Hepatitis C Infection

**DOI:** 10.1371/journal.pone.0027223

**Published:** 2011-12-02

**Authors:** Tetsuro Takayama, Hirotoshi Ebinuma, Shinichiro Tada, Yoshiyuki Yamagishi, Kanji Wakabayashi, Keisuke Ojiro, Takanori Kanai, Hidetsugu Saito, Toshifumi Hibi, for the Keio Association for the Study of Liver Diseases

**Affiliations:** Division of Gastroenterology and Hepatology, Department of Internal Medicine, Keio University School of Medicine, Shinjuku, Tokyo, Japan; Pohang University of Science and Technology, Korea, Republic of

## Abstract

Treatment with pegylated interferon alpha-2b (PEGIFN) plus ribavirin (RBV) is standard therapy for patients with chronic hepatitis C. Although the effectiveness, patients with high titres of group Ib hepatitis C virus (HCV) respond poorly compared to other genotypes. At present, we cannot predict the effect in an individual. Previous studies have used traditional statistical analysis by assuming a linear relationship between clinical features, but most phenomena in the clinical situation are not linearly related. The aim of this study is to predict the effect of PEG IFN plus RBV therapy on an individual patient level using an artificial neural network system (ANN). 156 patients with HCV group 1b from multiple centres were treated with PEGIFN (1.5 µg/kg) plus RBV (400–1000 mg) for 48 weeks. Data on the patients' demographics, laboratory tests, PEGIFN, and RBV doses, early viral responses (EVR), and sustained viral responses were collected. Clinical data were randomly divided into training data set and validation data set and analyzed using multiple logistic regression analysis (MLRs) and ANN to predict individual outcomes. The sensitivities of predictive expression were 0.45 for the MLRs models and 0.82 for the ANNs and specificities were 0.55 for the MLR and 0.88 for the ANN. Non-linear relation analysis showed that EVR, serum creatinine, initial dose of Ribavirin, gender and age were important predictive factors, suggesting non-linearly related to outcome. In conclusion, ANN was more accurate than MLRs in predicting the outcome of PEGIFN plus RBV therapy in patients with group 1b HCV.

## Introduction

Chronic hepatitis C (CHC) is of global concern because CHC patients frequently develop liver cirrhosis and hepatocellular carcinoma (HCC). Eradication of the hepatitis C virus (HCV) is an effective means of preventing CHC. Pegylated interferon alpha-2b (PEGIFN) plus ribavirin (RBV) combination therapy against the HCV is currently standard therapy for patients with CHC. Although this combination is effective against certain types of HCV, it is effective in only 50–60% of patients infected with the IFN-resistant strain of HCV [1]. HCV genotype 1 is common in the United States [2], Europe, and Japan. In Japan, 70% of CHC patients are infected with HCV genotype 1b [2]–[6]. The treatment outcome of patients infected with HCV genotype 1b is poor compare to other genotypes and the virus is eradicated from only 50% of these patients [7]–[11].

Although prolonged treatment with an elevated dose of RBV increases the efficacy of PEGIFN plus RBV treatment [12], the response rate is still relatively low. Furthermore, indices for determining whether to continue or stop treatment are lacking. Seventy-five % of patients treated with IFN experience systemic side-effects [1], the treatment of which adds to the cost and duration of IFN treatment. Therefore, it is important to identify factors predictive of treatment efficacy. Early viral response (EVR), a 2-log decrease in the serum HCV RNA level 12 weeks after commencing therapy, is a useful predictive factor. We also have demonstrated host and viral predictive factors [13]–[15].

Current guidelines recommend that treatment be discontinued for patients who do not achieve viral clearance from sera until 24 weeks after commencing therapy [1]; however, only 50–70% of patients achieve EVR [1]. Moreover, it is recommended that the decision to discontinue treatment should be made on an individual basis according to the patient's tolerance of therapy and biochemical or viral responses to treatment [1].

Previous studies, which typically used linear discriminant analysis provided the significant factors, though were unable to predict treatment outcomes at the level of the individual patient. Many clinical analyses have employed classical linear methods even though most data obtained in clinical settings are confounded and variables are not linearly related. A recent study demonstrated that the kinetics of most phenomena in living organisms are non-linear [16]. For these reasons, most data derived from clinical epidemiological or statistical studies are inappropriate for predicting responses at the level of the individual [16].

Artificial neural networks (ANNs) do not suffer from the problems inherent in traditional prediction methods. An ANN is a learning system based on a computational technique and has been used to simulate the neurological processing ability of the human brain [17]. ANNs recognise complex patterns between inputs and outputs via the learning process. Once the hidden relationship between input and output has been learned, an ANN can correctly predict output from a given input [18], [19]. ANNs are considered more suitable than MLRs for solving problems of the non-linear type and for analysing complex datasets [20]–[24]. Notably, ANNs can provide conclusive predictions at the individual level [16].

Previous reports have demonstrated that ANNs are superior to classical linear methods in the prediction of responses to interferon-α and RBV [20], [21], [23]–[26]. It is unclear whether the results of classical linear studies are representative of clinical conditions because all genotypes and a high number of responders were included in these studies. Moreover, liver biopsy results were often used as input data in classical linear studies. Although liver biopsies are useful, the procedure is associated with a high degree of risk and a large sampling error [27]. Alternative non-invasive and low-cost predictive methods are required.

The aims of this study were to develop a new model for predicting responses to PEGIFN plus RBV combination therapy in CHC patients infected with HCV genotype 1b by using clinical and laboratory data and an ANN and to identify factors that have non-linear relationships with responses.

## Results

### Response rate and patient backgrounds

One hundred and fifty-six patients (101 men and 55 women; mean age, 57.6 years; range of age, 18–77 years) received PEGIFN plus RBV therapy (table 1). Of the 156 patients, 66 patients (42.3%) achieved SVR.

**Table 1:** Characteristics of patients.

	Total n = 156
Mean age (range)	57.6 (18–77)
Sex	Male, 101; Female, 55
Weight	61.1 (39.4–99.5)
Height	163.7 (143.9–186)
Previous treatment	
Interferon	Yes: 67 (42.9%) No: 89 (57.1%)
Interferon plus RBV	Yes: 26 (16.7%) No: 130 (83.3%)
Initial dose of PEGIFN	87.1 (30–150)
Initial dose of RBV	668 (400–1000)
WBC	4884 (2300–9760)
RBC	456 (319–592)
Hb	14.4 (10.9–17.6)
Plt	16.6 (5.8–39.9)
AST	62 (20–246)
ALT	81 (15–309)
Cre	0.77 (0.47–1.40)
TC	177.6 (92–309)
Diabetes mellitus	Yes: 15 (9.6%) No: 124 (79.5%) Not determined: 17 (10.9%)
HCV RNA level	1842.1 (0.28–7774.1)
Total amount of PEGIFN (µg/kg/d)	1.15 (0.022–1.889)
Total amount of RBV (mg/kg/d)	8.27 (0.223–14.545)
SVR of HCV after 12 weeks	Yes: 80 (51.3%) No: 76 (48.7%)

Continuous data are expressed as the mean with the range or percentage in parentheses. WBC: white blood cell count, RBC: red blood cell count, Hb: serum haemoglobin, Plt: platelet count, AST: asparate aminotransaminase ALT: alanine transaminase, Cre: creatinine, TC: total cholesterol, SVR: sustained viral response, HCV: hepatitis C virus.

### Input factors and Outcome

We used the clinical data to determine input factors *X*_1_–*X*_21_, which were used to predict the outcomes of individual patients using MLR and ANN analysis (table 2). *X*_1_–*X*_4_ represented the patient's sex, age, height, and weight, respectively. *X*_5_ and *X*_6_ represented previous treatment with interferon and interferon plus RBV, respectively. *X*_7_ and *X*_8_ represented the initial doses of PEGIFN and RBV, respectively. *X*_9_–*X*_16_ represented laboratory variables (*X*_9_, white blood cell count; *X*_10_, red blood cell count; *X*_11_, haemoglobin level; *X*_12_, platelet count; *X*_13_, serum AST level; *X*_14_, serum ALT level; *X*_15_, serum creatinine level; and *X*_16_, serum total cholesterol level). *X*_17_ represented the presence of diabetes mellitus, *X*_18_ represented the HCV RNA level, *X*_19_ and *X*_20_ represented the total amount of administered PEGIFN and RBV, respectively, and *X*_21_ represented EVR, defined as the a 2-log decrease in the serum HCV RNA 12 weeks after therapy began. The outcome was SVR, which was determined 24 weeks after cessation of therapy.

**Table 2:** Factors and outcomes used to predict individual patient outcomes.

Factor	
*X*_1_: 1 = Male, 2 = Female	*X*_12_: Haemoglobin(g/dL)
*X*_2_: Age, 57.6±10.3	*X*_13_: Plt (×10^5^/mL)
*X*_3_: Height (cm)	*X*_14_: AST (IU/L)
*X*_4_: Weight (kg)	*X*_15_: ALT (IU/L)
*X*_5_: Previous therapy with interferon 0 = no, 1 = yes	*X*_16_: Diabetes mellitus 0 = no, 1 = not determined, 2 = yes
*X*_6_: Previous therapy with interferon plus RBV 0 = no, 1 = yes	*X*_17_: Serum total cholesterol level (mg/dL)
*X*_7_: Initial dose of PEGIFN (µg)	*X*_18_: HCV RNA level before treatment (kIU/mL)
*X*_8_: Initial dose of RBV (mg)	*X*_19_: Total amount of PEGIFN administered (µg/kg/week)
*X*_9_: Serum creatinine level (g/dL)	*X*_20_: Total amount of RBV administered (mg/kg/day)
*X*_10_: WBC level (/mL)	*X*_21_: EVR 0 = no, 1 = yes
*X*_11_: RBC level (×10^6^/mL)	
Outcome	
SVR 24 weeks after commencement of treatment 0 = no, 1 = yes	

### Significant factors for the prediction of SVR

For the prediction of SVR, factor *X*_21_ (EVR) was highly significant and had a high χ^2^ value (p<0.0001; table 3). Factor *X*_20_ (total amount of RBV administrated) was the next most effective factor (p<0.05). Factors *X*_1_ (sex), *X*_9_ (serum creatinine level), *X*_15_ (ALT level), and *X*_16_ (presence of diabetes mellitus) were the next most effective factors, but their regression coefficients were not statistically significant. Other factors had little effect on the response to therapy.

**Table 3:** Results of multiple logistic regression analysis.

Factor	Regression coefficient	Standarderror	X^2^ value	p value
Constant	3.8391	8.2907	0.21	0.6433
*X*_1_	0.9712	0.4989	3.79	0.0516
*X*_2_	0.0250	0.0263	0.90	0.3429
*X*_3_	−0.0117	0.0474	0.06	0.8045
*X*_4_	0.0334	0.0437	0.59	0.4439
*X*_5_	0.0349	0.2550	0.02	0.8911
*X*_6_	0.0216	0.3366	0.00	0.9489
*X*_7_	0.0118	0.0254	0.21	0.6438
*X*_8_	−0.0046	0.0031	2.24	0.1344
*X*_9_	−3.6052	2.0527	3.08	0.0790
*X*_10_	−0.0001	0.0002	0.24	0.6227
*X*_11_	0.0155	0.0100	2.39	0.1221
*X*_12_	−0.3129	0.3349	0.87	0.3503
*X*_13_	−0.0389	0.0549	0.50	0.4789
*X*_14_	0.0173	0.0149	1.35	0.2452
*X*_15_	−0.0151	0.0086	3.10	0.0785
*X*_16_ (0)	0.3585	0.4015	0.80	0.3719
*X*_16_ (1)	−1.1350	0.6219	3.33	0.0680
*X*_17_	−0.0024	0.0088	0.08	0.7812
*X*_18_	0.0007	0.0002	0.72	0.3970
*X*_19_	0.5059	0.9554	0.28	0.5965
*X*_20_	−0.2572	0.1136	5.12	0.0236
*X*_21_	1.4625	0.2725	28.80	<0.0001

### Non-linear relation exists between input factors and SVR

**Figure 1:**
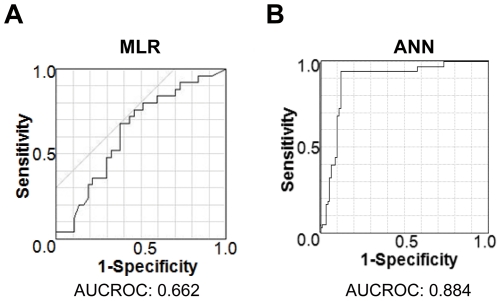
ROCs for multiple logistic regression models and ANN: (A) MLRs, (B) ANNs.

Next, we generated the predictive expression by using MLR and ANN to predict outcomes from multiple factors as determined by the aforementioned tests. We randomly divided the whole data into training data set for generation of the predictive expressions and validation data set to evaluate their accuracy (table 4). As shown in table 4, there were no significant difference in all factors between training data set and validation data set. The sensitivities were 0.45 in MLR and 0.82 in ANN (table 5). The specificities were 0.55 in MLR and 0.88 in ANN. The low frequency of both sensitivity and specificity in MLR and improved in ANN suggest that a non-linear relationship exists between inputs and outcomes. We also conducted ROC curve analysis to evaluate the accuracy of each prediction. To validate propriety, we analysed the ROC using validation data without training data. The area under the curves of the ROCs (AUROCs) of MLR was 0.662 and the mean AUROCs for ANNs was 0.884 (figure 1).

**Table 4:** Characteristics of patients in training and validation data set.

Total n = 156	Training n = 99	Validation n = 57
Mean age (range)	56.5	59.3
Sex	Male, 67; Female, 32	Male, 33; Female, 24
Weight	62.1	59.3
Height	164.1	162.9
Previous treatment		
Interferon	Yes: 40 No: 59	Yes: 27 No: 30
Interferon plus RBV	Yes: 19 No: 80	Yes: 7 No: 50
Initial dose of PEGIFN	85.8	89.5
Initial dose of RBV	662	677
WBC	4895	4864
RBC	459	452
Hb	14.4	14.3
Plt	16.1	17.4
AST	64	57
ALT	88	69
Cre	0.78	0.76
TC	176.3	1763
Diabetes mellitus	Yes: 8 No: 74 Not determined: 17	Yes: 7 No: 50 Not determined: 0
HCV RNA level	1957.7	1641.4
Total amount of PEGIFN (µg/kg/d)	1.11	1.22
Total amount of RBV (mg/kg/d)	8.57	7.79
SVR of HCV after 12 weeks	Yes: 51 No: 48	Yes: 29 No: 28

Data showed the mean or numbers of the factors.

No significant differences were exist in all factors between training data and validation data.

**Table 5:** The sensitivity and specificity provided by multiple logistic regression analysis and ANN.

	MLR	ANN
Sensitivity	0.45	0.82
Specificity	0.55	0.88

**Figure 2:**
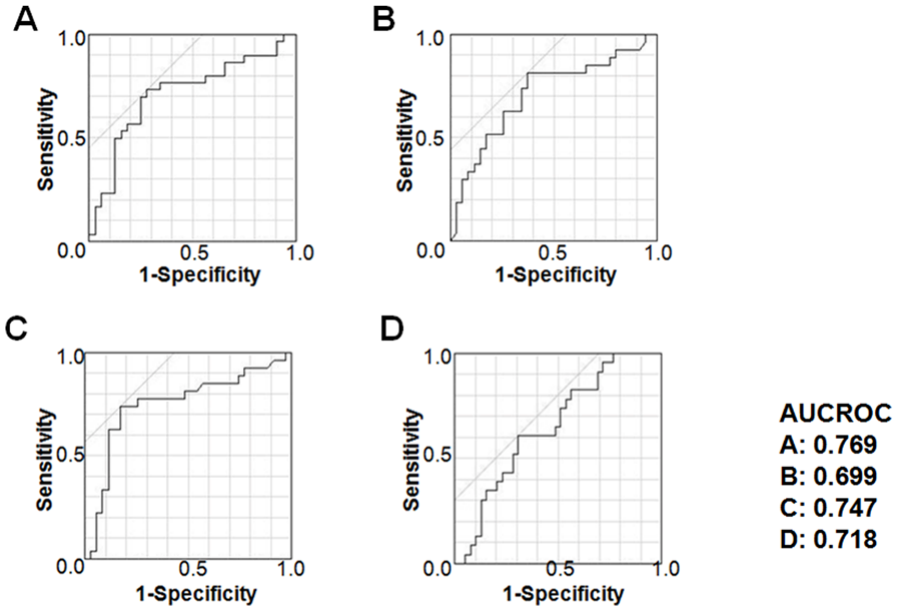
ROCs for four multiple logistic regression models: (A) trial 1, (B) trial 2, (C) trial 3, and (D) trial 4.

**Figure 3:**
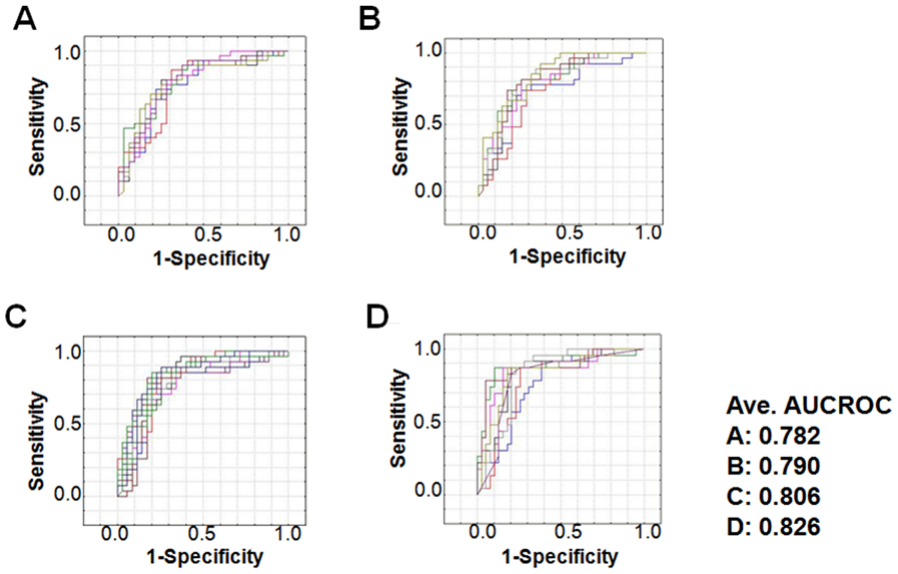
ROCs for several expressions of ANNs: (A) ANN1, (B) ANN2, (C) ANN3, and (D) ANN4. Several expressions were generated in each data set and the each lines show the different expressions of ANNs. Ave. AUCROC shows the average of expressions in each data set.

To evaluate the superiority in prediction of ANN, we randomly divided the all data into training data and validation data for 4 more times and evaluated the accuracy with same way. In all trials, the correct answer rate and AUROCs for the ANNs were significantly greater than that for MLRs (p<0.05) (table 6, 7 and figure 2, 3).

**Table 6:** The incidence of correct answers (%) provided by multiple logistic regression analysis.

	Trial 1	Trial 2	Trial 3	Trial 4
Correct answers (%)	72.6	64.5	75.8	67.7

**Table 7:** The incidence of correct predictions (%) provided by the ANN.

	ANN 1	ANN 2	ANN 3	ANN 4
Training data set	85.9	83.4	79.6	80.5
Validation data set	74.9	75.4	78.2	80.0

### Relative weights of the input factors

**Figure 4:**
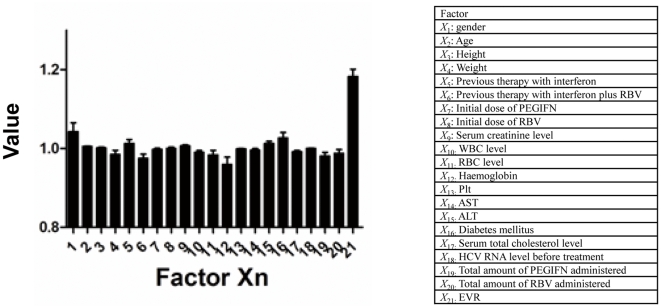
Non-linear analysis of factors for ANNs. Data are expressed as the mean±SD for member networks.

We analyzed relative weights of the input factors to identify factors that had a significant effect (including both linear and non-linear relationship) on the result of ANN (figure 4). Relative weights of the input factors determine how the result changes when the test factor (*X*_test_) is excluded. An *X*_test_ value greater than 1 indicates that it improves the expression, and a value less than 1 indicates that it does not improve the expression. We analysed the value of all networks and determined the corresponding means and standard deviations. *X*_21_: EVR was the most important predictive factor in every trial. The means of *X*_1_: gender and *X*_2_: age, *X*_3_: height, *X*_5_: previous therapy with interferon, *X*_8_: initial dose of Ribavirin, *X*_9_: serum creatinine, *X*_15_: ALT, *X*_16_: Diabetes mellitus, *X*_18_: HCV RNA level before treatment were also more than 1, which indicates that they have non-linear relationships with response to therapy.

### Impact of post-treatment factors in prediction of treatment

We also tried to predict the effect only using pre-treatment parameters (without using post-treatment parameters: *X*_19_: Total amount of PEGIFN administered, *X*_20_: Total amount of RBV administered, and *X*_21_: EVR), though the sensitivity and specificity were low (MLR; sensitivity 0.45, specificity 0.49, ANN; sensitivity 0.59, specificity 0.71) (table 8).

**Table 8:** The sensitivity and specificity provided by multiple logistic regression analysis and ANN without post-treatment parameters.

	MLR	ANN
Sensitivity	0.45	0.59
Specificity	0.49	0.71

## Discussion

Interactions between clinical, genetic, and environmental factors may affect the efficacy of PEGIFN plus RBV combination therapy in CHC patients and should be taken into account by physicians when interpreting indications for therapy. Although there are reports on predictors of the response to treatment against the HCV [28], [29], data derived from clinical epidemiology studies and medical statistics do not always result in correct predictions at the level of the individual patient. For instance, both male sex and low total cholesterol level are considered indicative of a good prognosis [28], but the prognosis for a male who also has a high total cholesterol level is unknown. In contrast, ANNs can identify relationships within a patient's clinical data that may be overlooked when classical linear approaches are used [16]. MLRs are powerful tool to find significant factors and provide the key factors in present our study, though it is not suit to predict the results by using factors non-linearly correlate. Because ANNs are trained using existing data, they are more capable of providing correct answers for individual patients. The ANN also has theoretical advantages over conventional MLRs. Unlike MLRs, ANN can predict both linear and non-linear phenomena and can analyse relationships between many variables at different levels [25].

The incidence of correct answers and the AUC of the MLRs differed greatly from that of the ANN. Moreover, it can say that data used in most previous studies were not validated because input data sets were used to estimate ROCs. Therefore, we used validation data sets to estimate ROCs. If we had used only input data in the ANN, the AUROC would have been equal to 100% because an ANN can fit input data perfectly. Compared with MLRs, a well-trained ANN can predict both linear and non-linear data.

We note that, although the ANN is a useful model, the network logic of prediction cannot be broken down into simple elements because ANNs process data in a non-linear way [16], [18], [25], [30]–[32]. We analysed the relative weights of input factors to address this issue. The values of each factor affecting the outcome was analysed (figure 2). EVR was identified as the most important factor. Serum creatinine, initial dose of Ribavirin, gender and age also had high values (figure 2).

Both physicians and patients express concern about the risks associated with treatment because the outcome is difficult to predict at the time decisions are made. The increased demand for individualised treatment necessitates new statistics that can be applied in conjunction with ethical and clinical evidence at the individual level. ANNs also have potential economic benefits in that they reduce unnecessary medical treatment.

A report on the classification of patients was published recently [33]. Although this is a valid strategy, it is difficult to apply under clinical conditions because the ISDR mutant and Th1∶Th2 ratio must first be determined. Moreover, there are some conflicting reports on the ISDR mutant [34]. As the aforementioned report did not performed validation, they should not be compared with our results; however, the predictive accuracy of our technique is superior to them.

The predictive expression developed in this study should aid physicians to advise individual patients on whether to continue with PEGIFN plus RBV combination therapy. We also tried to predict the effect only using pre-treatment parameters (table 8). Compare to the table 5, both sensitivity and specificity were dramatically improved by adding post-treatment parameter. Suggesting, post-treatment parameter such as adherence to treatment might affect to the effect of PEGIFN plus RBV combination therapy. As the EVR and total amount of RBV were the most important parameters in our study, the predictive expression could also be used to determine whether to increase the dose of RBV. Because we included the total amount of PEGIFN and RBV in the data sets, the effect of an increased dose can be simulated. Although the magnitude of the dose effect depends on patient's symptoms and exposure to adverse events, our technique remains a powerful tool for determining the appropriate dose of PEGIFN and RBV.

Although our predictive expression does not predict responses perfectly, our results show that the ANN is a valid method for devising individual treatment regimens in the clinical situation. It is well known that 100% prediction accuracy is impossible to achieve because of random error and multiple biases.

As the outcome of PEGIFN plus RBV treatment may be affected by multiple unknown factors, it is important to update data continuously and to acquire clinical data such as the patient's demographics, medical history, and laboratory test results. Recent accumulating data revealed the importance of *IL28B* gene from genome wide study [35]–[37]. Especially, very recent data clearly showed the significance of SNP rs12979860 in *IL28B* gene in the prediction of the treatment outcome [38]–[41]. We could not assess the effect of them in this study since we have not collected those data. Further analyses were needed though it may be improve the accuracy. It is also important to demonstrate that the use of trained ANNs in routine medical practice increases the quality of medical care and reduces costs.

## Methods

### Patients

The study was conducted by the Keio Association for the Study of Liver Disease (Supporting Information S1). This study was approved by the Keio University School of Medicine review board and the permission was obtained (ID number 2010-026). One hundred and fifty-six CHC patients (101 men and 55 women; mean age, 57.6 years) infected with genotype 1b HCV and treated with PEGIFN plus RBV combination therapy from December 2004 to May 2007 who had been assessed for sustained viral response (SVR) were enrolled and the data were collected. SVR was defined as an absence of serum HCV RNA 24 weeks after cessation of therapy.

All patients had HCV genotype 1b and HCV RNA levels in excess of 100 kIU/mL as measured by quantitative Cobas Amplicor assays (Roche Diagnostics Co. Ltd, Tokyo, Japan). Exclusion criteria were pregnant women or women of childbearing potential, nursing mothers, male patients whose partner could have become pregnant, anaemia, leucopenia, thrombocytopenia, severe dysfunction of organs other than the liver, infection with hepatitis B virus or human immunodeficiency virus, autoimmune hepatitis, primary biliary cirrhosis, and liver dysfunction caused by drugs.

Some of the patients did not undergo a liver biopsy because not all of the centres could perform biopsies. All patients were treated for 48 weeks and were followed up for 48 weeks after treatment.

The purpose of the study and its protocol were explained to all patients and their written, informed consent was obtained.

### PEGIFN plus RBV combination therapy

PEGIFN-α2b (Schering–Plough K.K., Tokyo, Japan) was administered weekly in doses adjusted for body weight according to the manufacturer's recommendations in Japan (45 kg or less, 60 µg; 46–60 kg, 80 µg; 61–75 kg, 100 µg; 76–90 kg, 120 µg; and 91 kg or more, 150 µg). RBV (Schering–Plough K.K.) was administered once daily in doses adjusted for body weight according to the manufacturer's recommendations in Japan (61 kg or less, 600 mg; 61–80 kg, 800 mg; and 81 kg or more, 1000 mg).

The duration of PEGIFN plus RBV therapy was 48 weeks and patients were followed-up for the subsequent 48 weeks. Serum levels of HCV RNA were quantified by amplicor analysis. Blood was analysed at the beginning of treatment and every 4 weeks thereafter.

A questionnaire was used to review demographic data (age, sex, weight, and height), previous treatment, initial dose of PEGIFN, initial dose of RBV, presence of diabetes mellitus, HCV RNA level before therapy, amount of PEGIFN and RBV administered, SVR, and serum concentrations of white blood cells (WBCs), red blood cells (RBCs), platelets (Plts), asparate aminotransaminase (AST), alanine transaminase (ALT), creatinine (Cre), and total cholesterol (TC).

As the data were collected from several centres in various prefectures, within-centre bias was excluded.

### ANN

To develop the ANN, we used three types of network according to manufacturer's instruction: multilayer perceptrons (MLPs), radial-basis function networks (RBFs), and linear networks (LINs). Details of the ANN and MLP are provided elsewhere [30]. In brief, a hierarchical ANN consisting of three layers (one input, one hidden, and one output layer) was used to classify the effect as a node in the output layer. MLPs were constructed from three layers (one input, one hidden, and one output layer) to classify effects as a node in the output layer. RBF units respond to the distance of points from the centre. The RBF has a hidden layer of radial units, each of which models a Gaussian response surface. We analyzed the results of 156 patients from multiple centres and formed 100 000 networks.

### Training data set and validation data set

We used same training data set for generating the predictive expression by using MLR and ANN, and used validation data set to evaluate the accuracy of the expression generated using training data set.

### Data analysis

Accuracy (correct answer rate), sensitivity, and specificity were calculated. Also receiver-operating characteristic (ROC) curves for the MLR and ANN were generated to evaluate their accuracy [25]. Multiple logistic analysis was performed using JMP version 7.0.1 software (SAS Institute Japan, Co., Ltd, Tokyo, Japan) and ANN was analysed using Statistica version 06J software (StatSoft Japan, Co., Ltd, Tokyo Japan).

### Relative weights of input factors analysis

The detail of relative weights of input factors analysis ( = sensitivity analysis) were described elsewhere [42]. In brief, we analysed relative weights of input factors using a leave-one-input-factor-out (LOFO) in turn with a missing values substitution procedure, which enables predictions to be made in the absence of values for each causal factor, and then assessed effects upon ANN response error. Root mean square error (RMSE) for prediction is defined as:
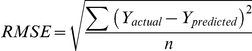
where, *n* is the number of validation data. Y_actual_ and Y_prediced_ are the outcomes of actual values and predicted ones, respectively. RMSE is an estimate of the typical difference between the predicted and actual values of outcomes. The smaller RMSE the better the prediction accuracy of the models is.

The network original error was accumulated as RMSE_original_ and the network was again used with LOFO data and the error RMSE_LOFO_ was estimated. Then, the relative weights of input factors was calculated as RMSE_LOFO_/RMSE_original_.

## Supporting Information

Supporting Information S1 (DOC)
