# Supplementary material for: Prediction of Effect of Pegylated Interferon Alpha-2b plus Ribavirin Combination Therapy in Patients with Chronic Hepatitis C Infection
Source: PLoS One. 2011 Dec 2;6(12):e27223. doi: 10.1371/journal.pone.0027223 (PMC3229481; doi:10.1371/journal.pone.0027223)
Supplement: Supporting Information S1 — (DOC) [file pone.0027223.s001.doc]

**Supporting Information**

The other Keio Association for the Study of Liver Diseases group is consisted of followings: Drs. Yasutaka Inagaki, Tetsuya Masuda (Nihon Kohkan Hosipital, Kanagawa), Drs. Yoshinori Horie, Masahiro Kikuchi (Eiju General Hospital, Tokyo), Drs. Nobuhiro Tsukada, Norio Maeda (Tokyo Metropolitan Saisei-kai Central Hospital, Tokyo), Drs. Naoki Kumagai, Satoshi Tsunematsu, Kanji Tsuchimoto (Kitasato Institute Hospital, Tokyo), Dr Kazuo Kasiwazaki (Tachikawa Hospital, Tokyo), Dr. Masahiko Takahashi (Tokyo Metropolitan Hiroo Hospital, Tokyo), Dr. Kazuhiro Atsukawa (Hiratsuka Municipal Hospital, Kanagawa), Dr. Jiro Nishida (Tokyo Dental Collage Ichikawa General Hospital, Chiba).
